# Patient-reported outcomes with subcutaneous immunoglobulin in secondary immunodeficiency

**DOI:** 10.3389/fimmu.2025.1528414

**Published:** 2025-03-20

**Authors:** Juthaporn Cowan, Il-Kang Na, André Gladiator, Marta Kamieniak, S. Shahzad Mustafa

**Affiliations:** ^1^ Department of Medicine, Division of Infectious Diseases, University of Ottawa, Ottawa, ON, Canada; ^2^ Department of Biochemistry, Microbiology and Immunology, and Centre for Infection, Immunity and Inflammation, University of Ottawa, Ottawa, ON, Canada; ^3^ Inflammation and Chronic Disease Program, The Ottawa Hospital Research Institute, Ottawa, ON, Canada; ^4^ Department of Hematology, Oncology and Tumor Immunology, Charité – Universitätsmedizin Berlin, Corporate Member of Freie Universität Berlin, Humboldt‐ Universität zu Berlin, and Berlin Institute of Health, Berlin, Germany; ^5^ Experimental and Clinical Research Center, A Cooperation of Charité-Universitätsmedizin Berlin and Max Delbrück Center for Molecular Medicine, Berlin, Germany; ^6^ Berlin Institute of Health (BIH) at Charité, Universitätsmedizin Berlin, BIH Center for Regenerative Therapies (BCRT), Berlin, Germany; ^7^ German Consortium for Translational Cancer Research, (DKTK), Partner Site Berlin, Charité, Universitätsmedizin, Berlin, Germany; ^8^ Global Medical Affairs, Takeda Pharmaceuticals International AG, Zurich, Switzerland; ^9^ Global Medical Affairs, Takeda Development Center Americas, Inc., Cambridge, MA, United States; ^10^ Division of Allergy, Immunology, and Rheumatology, Rochester Regional Health, Rochester, NY, United States; ^11^ Department of Medicine, University of Rochester School of Medicine and Dentistry, Rochester, NY, United States

**Keywords:** secondary immunodeficiency, hematological malignancy, subcutaneous immunoglobulin, patient-reported outcomes, health-related quality of life, treatment satisfaction, patient preference, shared decision-making

## Abstract

Subcutaneous (SCIG) and intravenous immunoglobulin (IVIG) replacement are both used to prevent infections in patients with secondary immunodeficiency (SID). Compared with IVIG, SCIG has fewer systemic side effects and, additionally, facilitates home-based treatment. Shared decision-making practice should include discussion of aspects such as patient preference as well as the associated risks and benefits of treatment. We review the available evidence for the use of SCIG treatment in patients with SID, focusing on patient-reported outcomes (PROs). In most studies, there were improvements to health-related quality of life with SCIG treatment, compared with before initiating SCIG without prior IVIG treatment, or after switching to SCIG from IVIG treatment, or a no-SCIG/IVIG cohort. Treatment satisfaction with SCIG was similar between patients with SID and primary immunodeficiency disease. Patient preference and perception assessments highlighted the benefits of SCIG compared with IVIG, such as ease of use and administration, convenience, and time-effectiveness. In addition, many patients self-administered SCIG at home. Such aspects may be of specific benefit to patients with SID and hematological malignancy by reducing the risk of infection exposure in clinical settings. PRO data may be useful during shared decision-making discussions with patients with SID.

## 1 Introduction

Secondary immunodeficiency (SID) may occur because of deficient antibody production or antibody loss due to various diseases and their associated treatment (for example, hematological malignancy and immunosuppressive medical treatment) as well as medications and surgical procedures (1–3). Patients with SID and B cell malignancy are susceptible to recurrent, severe, or opportunistic infections, which are a significant cause of morbidity and mortality (4–6). Older patients with hematological malignancy and comorbidities, such as hypertension, cytopenia, and obesity (4, 7), are more likely to develop complications correlated with SID than younger patients (8) without similar comorbidities. These patients may be undergoing concomitant cancer treatment and have difficulties with venous access (9–11). Emerging therapies for hematological malignancies, such as chimeric antigen receptor (CAR) T-cell and bispecific antibody therapies, may also increase this risk of infection in this patient population (12).

Immunoglobulin replacement therapy (IgRT) is used to prevent recurrent or severe infection in patients with primary immunodeficiency (PID) or SID (1, 3). In hematological malignancies, guidelines recommend that IgRT may be considered as a supportive treatment for patients with SID and recurrent or severe infections, after prophylactic vaccination (with non-live vaccines) and/or antibiotics (1, 13, 14). In addition, intravenous immunoglobulin (IVIG) is recommended for prophylactic use in patients with multiple myeloma treated with CAR T-cell, or bispecific antibody therapies with an immunoglobulin G (IgG) level of < 4 g/L and/or high infection risk (12, 15–17). In patients with hematological malignancy-associated SID, IgRT treatment is well tolerated, reduces the rate of severe infections, decreases use of antibiotics and hospital admissions, and improves quality of life (8, 9, 18, 19). Despite SID being more prevalent than PID, the body of evidence for the use of IgRT is larger for PID than for SID (3, 20).

IgRT can be delivered by IVIG or subcutaneous (SCIG) infusion (3, 20). Clinical trials of IVIG in the 1980s and 1990s provide the majority of efficacy and safety data for IgRT in SID (3, 18, 21). IVIG administration requires venous access and is generally given under medical supervision every 3–4 weeks, due to the serum half-life of IgG (22). SCIG formulations have since been developed and approved for use in patients with SID and are typically administered up to every 2 weeks (22, 23). With appropriate patient/caregiver training, SCIG allows for flexible home-based treatment schedules with no venous access requirement and may therefore suit some patients with SID, who would otherwise require multiple outpatient visits (9). SCIG infusion parameters, such as method of administration (infusion pump or manually via syringe), infusion volume/rate, and number of sites/infusions can be adjusted according to patient preference (24). SCIG has a lower systemic adverse event (AE) profile than IVIG, although local AEs such as infusion-site reactions are more common (22). Additionally, the SCIG route shows lower fluctuations in serum IgG levels than IVIG, and therefore is associated with a lower risk of renal failure, hemolysis, or thrombosis (2, 25).

The conventional SCIG therapies available for the treatment of SID are shown in **Table 1**; data on hyaluronidase-facilitated SCIG 10% (HyQvia, Baxalta Innovations GmbH, a Takeda company, Vienna, Austria) are available but the focus of this review is conventional SCIG. As the evidence for the use and effectiveness of SCIG replacement in SID increases, product labels are being updated to reflect appropriate use in SID, rather than in hypogammaglobulinemia secondary to specific etiologies. For example, there was a label expansion in 2024 for immune globulin subcutaneous (human) 20% solution stabilized with glycine (IgGly20; Cuvitru [Baxalta US, Inc., a Takeda company, Lexington, MA, USA]) to include patients with SID experiencing severe or recurrent infections, ineffective antimicrobial treatment, or either proven specific antibody failure (failure to mount at a least a 2-fold rise in IgG antibody titer to pneumococcal polysaccharide and polypeptide antigen vaccines) or serum IgG < 4 g/L (30, 43).

**Table 1:** Conventional subcutaneous immunoglobulin formulations indicated for patients with SID.

Drug name	Concentration, grams Ig/100 mL, % (available vial sizes, mL)^a^	Indicated etiology of SID (including regional/country specific differences in terminology used)
Cutaquig	16.5% (6, 10, 12, 20, 24, 48 mL)	SID^b^ (26, 27)
Cuvitru (IgGly20)	20% (5, 10, 20, 40, 50 mL)	Symptomatic secondary hypogammaglobulinemia (28) Secondary humoral immunodeficiency (29) SID^b^ (30, 31)
Evogam	16% (5, 10, 20 mL)	Symptomatic secondary hypogammaglobulinemia (32, 33)
Gammanorm^c^	16.5% (6, 10, 12, 20, 24, 48 mL)	Hypogammaglobulinemia in patients with CLL, MM, or pre-/post-HSCT (34)
Hizentra (IgPro20)	20% (5, 10, 20, 50 mL)^d^	Symptomatic secondary hypogammaglobulinemia (35, 36) SID^b^ (37–39)
Subgam	16% (5, 6.25, 10, 12.5, 25 mL)	Hypogammaglobulinemia in patients with CLL, MM, or pre-/post-HSCT (40)
Xembify	20% (5, 10, 20, 50 mL)	Symptomatic secondary hypogammaglobulinemia (41) Hypogammaglobulinemia in patients with CLL, MM, or pre-/post-HSCT (42)

Table content based on product labels as of March 2025. Please refer to the most current product label or official sources for the latest updates and information.

a Not all vial sizes are available in all regions.

b Indicated in Europe and the UK for the treatment of SID in patients who suffer from severe or recurrent infections, ineffective antimicrobial treatment and either proven specific antibody failure (a failure to mount at least a 2-fold rise in IgG antibody titer to pneumococcal polysaccharide and polypeptide antigen vaccines) or serum IgG level of < 4g/L.

c Gammanorm is now discontinued in some countries.

d Also available in prefilled syringes of 5, 10, 20 and 50 mL.

CLL, chronic lymphocytic leukemia; HSCT, hematopoietic stem cell transplantation; Ig, immunoglobulin; MM, multiple myeloma; SID, secondary immunodeficiency.

Given that the efficacy of both IVIG and SCIG products are similar for most conditions, the choice of IgRT treatment should consider patient preference. Shared decision-making is influenced by patient preference, experience, and perception of treatment, and facilitates patient-centric care, leading to a better understanding of treatment risks/benefits, and ideally, improved adherence to treatment (13, 14, 44–47). Studies in patients with PIDs have demonstrated a patient/caregiver preference for home-based SCIG over IVIG and as well as an improvement in patient-reported outcomes (PROs) (48–50).

This publication reviews the evidence for the use of conventional SCIG treatment in patients with SID, with a focus on patient-centric outcomes such as health-related quality of life (HRQoL), treatment satisfaction, and patient preference, as well as infusion characteristics and infection-related outcomes. Within the context of these findings, we discuss the potential impact of outcomes with SID on shared decision-making practices.

## 2 Characteristics of patients with SID and SCIG infusions

We present findings from 11 predominantly real-world evidence studies that used PROs to assess treatment of SID with SCIG (EMBASE and Medline searched May 1, 2024, using free-text terms for SID, SCIG and PROs; see **Supplementary Materials Table S1** for search strategy and terms and **Supplementary Figure S1** for study selection) (9, 51–60). Three of these studies also reported data from patients with PIDs; here, we present results for patients with SID only, although any comparisons between patients with PIDs are noted. An overview of patient and infusion characteristics for each study is presented in **Supplementary Table S2**.

## 3 Patient-reported outcomes

A variety of PRO instruments have been used to compare treatments. The 36-item Short Form Health Survey (SF-36) is a multiscale survey which assesses HRQoL (61). The Treatment Satisfaction Questionnaire for Medication (TSQM-9) and Life Quality Index (LQI), assessed on 5 or 7 point Likert scales, were scored by domain and transformed into an overall score ranging 0–100, for which a higher score indicates higher treatment satisfaction (62, 63). SF-36 was used in four studies (52, 56–58); TSQM-9 was used in two studies (52, 55); LQI and a 13-item treatment preference questionnaire (TPQ) were used in the CANCUN study (patients were asked to rate their treatment experience on a 5-point scale ranging from “I like it very much” to “I dislike it very much”) (55). The other studies either used study-specific questionnaires (9, 51, 59, 60) or did not report the instruments used (53, 54). Variation in the types of PRO data available limited the comparisons between studies, but some trends in HRQoL and patient satisfaction and preference could be noted.

### 3.1 Health-related quality of life

**Figure 1:**
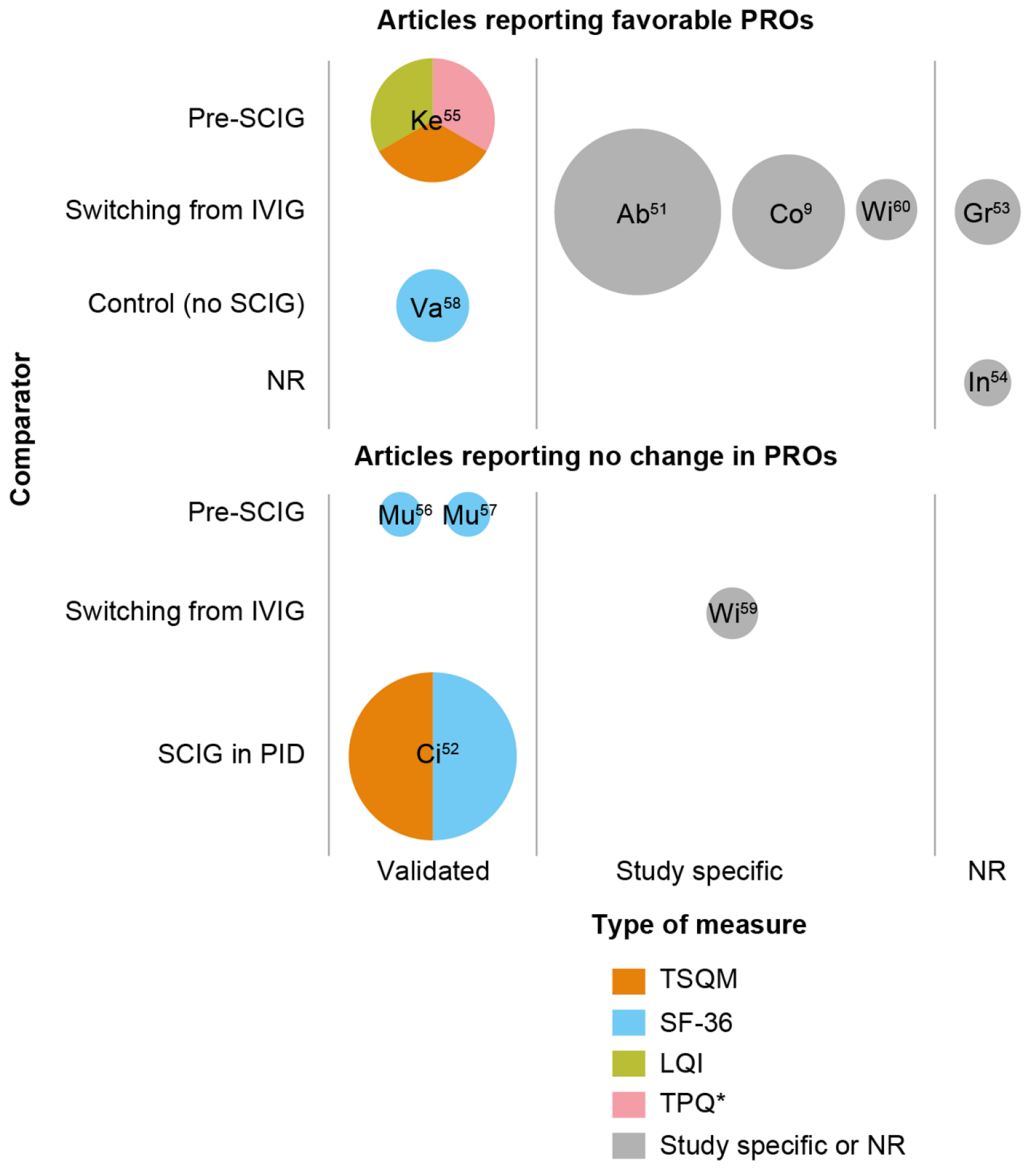
Studies including patient reported outcomes in patients with SID receiving conventional SCIG treatment. *TPQ used in this study was not a validated measure. Bubble size is indicative of the number of patients with SID who received SCIG. PRO data may not be available for all patients with SID who received SCIG in the study. Labels within the bubbles indicate the study and reference; see **Supplementary Table S1** for further information on PROs in individual studies. LQI, Life Quality Index; NR, not reported; SCIG, subcutaneous immunoglobulin; SF-36; 36-item Short Form Health Survey; TPQ, treatment preference questionnaire; TSQM-9, Treatment Satisfaction Questionnaire for Medication.

HRQoL was assessed using SF-36 (four studies) or other measures (five studies), including study-specific questionnaires (**Figure 1**). Of those using SF-36, one study reported significant improvements in HRQoL in the SCIG cohort compared with the no-SCIG cohort across the domains of general health, physical function, limitations in usual role activities due to physical/emotional problems, vitality, social function, and mental health (*p* < 0.05); the SF-36 score for the incidence of pain was numerically lower in the SCIG cohort than in the no-SCIG cohort, but the difference was not statistically significant (58). Two studies reported no significant change in SF-36 score during the 52 weeks in which patients received Ig20Gly (56) or 24 weeks of IgPro20 (57) (**Supplementary Table S2**); the authors of both studies noted that SCIG had been used prophylactically and that the patients generally had high HRQoL at baseline, which may have accounted for these results (56, 57). One study noted that HRQoL scores in PID and SID were similar (52).

Of the studies using other measures to assess quality of life, five were in patients switching to SCIG from IVIG. In one study, patients with SID reported that there were no improvements in health and HRQoL between IVIG and SCIG treatments (assessed via questions on patient-perceived side effects, treatment satisfaction, and impact of IgRT on social life, family life, work, and study, and overall quality of life) (59). By contrast in another study, SCIG was associated with an improvement in HRQoL in 33 patients who switched from IVIG to SCIG (SCIG 16% and SCIG 20% [IgPro20]): 33.3% felt that their health relating to infectious events was “much” or “somewhat better” with SCIG; 54.5% felt that it was “about the same”; 75.8% felt that their health relating to adverse events was “much” or “somewhat better”; and 15.2% felt it was “about the same” (9). The study also found an increase in the proportion of patients who felt that adverse events after IgRT did not interfere with their work life, from 54.4% during IVIG treatment to 69.7% during SCIG treatment, and when considering all aspects of infections, adverse events, and the possibility of home-infusion, 78.8% of patients reported the impact of SCIG on their health to be “much” or “somewhat better” than IVIG (9). Windegger et al. (2021) evaluated various outcomes including patient perception of SCIG treatment in patients with SID using a study-specific questionnaire; although fluctuations were reported over the observation period, quality of life (QoL) improved in 75% of patients in the first year after switching from IVIG to SCIG, coincident with an overall reduction in severe infections (60). Grywalska and Rolinski reported that SCIG 16.5% treatment resulted in significant improvements in QoL after a switch from IVIG treatment, but did not describe the measure used (53). In Innocenti et al., in which the HRQoL assessment was not described or directly compared between SCIG and IVIG, all patients reported “a benefit on QoL” during treatment with SCIG, owing to the flexibility of self-administration at home and the generally shorter time needed to perform an infusion versus IVIG (54). In the Ontario Immunoglobulin Treatment (ONIT) case registry study, 27 patients switched from IVIG to SCIG (SCIG 20% [IgGly20 or IgPro20] or SCIG 16.5%) and responded to the questionnaire: 62.9% reported their overall health status to be better after switching to SCIG and 33.3% reported overall health status to be the same (51).

### 3.2 Treatment satisfaction

Two studies reported that treatment satisfaction measured by TSQM-9 was comparable between patients with PID and patients with SID receiving SCIG (IgGly20 in the CANCUN study; SCIG 16%, SCIG 16.5%, SCIG 20%, and hyaluronidase-facilitated SCIG 10% in Cinetto et al.) (52, 55). In the CANCUN study, TSQM-9 scores indicated generally high satisfaction of patients with SID after 12 months of SCIG; the mean (standard deviation; SD) score was highest for the domains of effectiveness and global satisfaction – 79.8 (14.5) and 79.5 (16.3), respectively – and was 74.4 (14.9) for convenience (55). The mean TSQM-9 scores were not reported in the other study (52).

The mean (SD) LQI scores in the CANCUN study were also indicative of a high level of satisfaction with Ig20Gly: 95.3 (8.0) for therapy setting, 93.3 (7.7) for treatment interferences, 92.1 (12.0) for treatment costs, and 87.4 (9.6) for therapy-related problems (55).

### 3.3 Patient preference

In the CANCUN study, all patients with SID who completed the TPQ expressed an interest in continuing treatment with IgGly20 (55). Individual aspects of IgGly20 treatment which were most highly rated (either “I like it very much” or “I like it”) were convenience in general (96.2%), the option of self-administration (94.3%), the option to adjust own treatment schedule (94.3%), and ease of administration (73.6%). In contrast, the lowest rated aspect in relation to IgGly20 administration (“I dislike it”) was the number of punctures per month (9.4%); no patients reported “I dislike it very much” for any aspect in the TPQ (55). Overall, 73.6% (n = 39) of the respondents received treatment at home (55).

## 4 Efficacy and safety

Efficacy and safety are important attributes to consider when selecting treatment options. Efficacy of SCIG treatment has been assessed by monitoring IgG levels and infection-related outcomes (**Supplementary Table S2**). All studies reported favorable outcomes relating to IgG levels with SCIG: improvements in IgG levels from baseline while receiving SCIG (51, 54, 56, 57); higher IgG trough levels in a SCIG cohort compared with a no-SCIG control cohort (58); similar IgG levels between SID and PID cohorts (52, 55); or higher IgG levels with SCIG than with IVIG (SCIG and IVIG dosages/IgG levels are presented in **Supplementary Table S3**) (9, 53, 59, 60). There was a range of findings for infection-related outcomes. Six studies reported a reduction in infection rate for patients on SCIG compared with the period before SCIG, or compared with IVIG or a no-SCIG control group (9, 51, 53, 56–58). Four studies reported that both IVIG and SCIG were effective in reducing the incidence of infections (9, 56–58). In one study, after initiating SCIG, the proportion of patients in which no infection was reported increased throughout the study (12% in year 1 to 43% in year 3) and the mean (SD) infection rate per patient declined from 2.06 (1.52) in year 1 to 1.65 (2.23) in year 3 (60). Another study reported that no patients experienced infectious events while on SCIG therapy (54). One study reported that the annual rate of infections (2.15 vs 1.62, respectively), along with mean serum IgG trough levels, were higher in patients receiving SCIG than when receiving IVIG (59). Two studies reported a reduction in antibiotic use with SCIG compared with IVIG/no IgRT, or vs a control arm (9, 58), and two studies reported a decreased reliance on antibiotics during SCIG compared with pre-SCIG (56, 57).

Safety during SCIG treatment has been mostly assessed via AE monitoring (**Supplementary Table S2**). Overall, most AEs reported were local and mild, and four studies reported no systemic AEs during SCIG treatment (9, 56, 57, 60). When reported, SCIG treatment was generally well tolerated (9, 54, 56). Studies in which SCIG was compared with IVIG reported a more favorable safety profile for SCIG than for IVIG (9, 59); in one study, patients who received SCIG had no systemic or clinically relevant AEs and experienced fewer AEs than those who received IVIG (9).

## 5 Discussion

The primary focus of this literature review was patient-reported outcomes with conventional SCIG use in patients with SID in a real-world setting. Many studies that report PROs with IgRT do not separate data for patients with PIDs from those with SID (or the underlying condition), or separate data for SCIG from IVIG; consequently, the number of studies specific to our objective was limited and included congress abstracts. The studies reviewed were often small in size, and few reported PROs as a primary objective; accordingly, the studies were typically not designed for the purpose of exploring PROs. However, many findings of this review in SID are consistent with the larger body of evidence in PIDs.

Conventional SCIG used in real-word settings was delivered using 20%, 16%, and 16.5% solutions and hyaluronidase-facilitated 10% solution (9, 51, 52, 60, 64–66) and was most often given weekly with doses about 0.1 g/kg/week. When SCIG was compared with IVIG, the equivalent monthly doses were similar (51, 59, 60). Five studies reported that patients received instruction and then administered SCIG independently (9, 54, 56, 57, 60). These findings align with other studies in patients with PID, which have demonstrated that better SCIG training and efficient infusions correlate to more favorable PRO scores (64). Improved PRO scores were noted after switching from IVIG to SCIG (9, 51, 60), as has been reported for patients with PIDs (66). Although further studies specific to patients with SID are warranted, these findings help to bridge the gap in evidence between patients with PIDs and those with SID.

In the CANCUN study, 9.4% of participants reported that they disliked the number of punctures per month with SCIG (55). This may be partially addressed by hyaluronidase-facilitated SCIG 10%, in which co-administered hyaluronidase temporarily increases the permeability of subcutaneous tissue to facilitate the dispersion and absorption of immunoglobulin, allowing larger volumes, and therefore requiring less frequent infusions, than conventional SCIG (67, 68). The PRO data also demonstrate the positive attributes of SCIG for patients with SID, including ease of use and administration, convenience, time-effectiveness, and improved HRQoL compared with IVIG treatment, and align with findings for patients with PIDs for whom these characteristics are associated with higher treatment satisfaction (9, 51, 54, 55, 58, 60, 64, 65). For patients with PIDs, infection rate is lower for IgRT administration at home than in a hospital setting (69). Factors such as ease of use, home-based treatment, and no need for venous access may be particularly relevant for patients with SID who require IgRT.

When the condition underlying SID was recorded, every study reported hematological malignancies, with CLL often being the most common. The mean age of patients with SID ranged from 63–71 years (58, 59) and in the two studies that compared ages for SID with PID, patients with SID were older (mean age 69 vs 48 years and median age 69 vs 57 years) (52, 55). Home-based IgRT may be beneficial to an older patient population, and particularly those requiring cancer treatment. As well as the potential benefits to infection-related outcomes with SCIG use there are benefits to HRQoL: patients showed a strong appreciation for the ease of administration, autonomy of self-administration at home, and the ability to be able to adjust treatment to their own schedule (55).

Although efficacy and safety outcomes were not the focus of this review, they have the potential to influence patient experience/patient-centric outcomes and were reported in most of the studies included in this review. Many studies reported an improvement in IgG levels for patients with SID receiving SCIG (compared with baseline, IVIG, or a no-SCIG control), with the levels remaining above 5 g/L. The safety data reported were consistent with the existing evidence in PID that SCIG is generally well tolerated with a favorable safety profile (70, 71). Studies which compared safety outcomes in both SCIG and IVIG reported that although some patients receiving SCIG therapy experienced infusion-site reactions, SCIG was associated with a lower frequency of systemic adverse events (9, 59).

The PRO data reviewed here could be considered when approaching shared decision-making discussions with patients with SID. The advantages and disadvantages of each treatment modality (IVIG or SCIG), as well as attributes such as frequency, duration, and location of treatment, the number of needlesticks required, and patient preference should be discussed with patients with immunodeficiency (13, 47). Whilst IVIG may be a practical option if patients are already attending an outpatient infusion center for cancer treatment, the advantages of SCIG include convenience, autonomy, home-based treatment, and ease of administration (55). The use of supervised training sessions, which include in-person demonstrations and support from specialist teams, can be valuable in building patients’ confidence before self-administering SCIG at home.

In conclusion, patients reported improved HRQoL and ease of use during SCIG treatment, compared with no IgRT or IVIG. Together with existing efficacy and safety data, SCIG treatment offers benefits to patients with SID and should be considered during shared decision-making discussions with this population.
