# Supplementary material for: Patient-reported outcomes with subcutaneous immunoglobulin in secondary immunodeficiency
Source: Front Immunol. 2025 Mar 20;16:1528414. doi: 10.3389/fimmu.2025.1528414 (PMC11967276; doi:10.3389/fimmu.2025.1528414)
Supplement: Supplementary file 1 [file SupplementaryFile1.docx]

# **Supplementary materials**

## ***Table S1. Search strategy and terms used in database searching***

| **Embase** | | | |
| --- | --- | --- | --- |
| **#** | **Searches** | **Results** | **Topic** |
| 1 | (secondary immuno?deficien* or SID or (secondary adj2 deficienc*) or secondary hypogammaglobulinemia).mp. | 9543 | Advanced |
| 2 | (subcutaneous immunoglobulin or SCIG or cuvitru or hizentra or subcuvia or vivaglobin or cutaquig or evogram or gammagard or gamunex or gamastan or gammaked or gammanorm or subgam).tw. | 2680 | Advanced |
| 3 | (patient reported outcome* or PRO or self reported outcome* or quality of life or health related quality of life or qol or hrql or hrqol).ti,ab. | 1058091 | Advanced |
| 4 | (treatment preference or patient preference or preference for treatment or preferred treatment).ti,ab. | 20276 | Advanced |
| 5 | (treatment satisfaction or patient satisfaction or satisfaction with treatment).ti,ab. | 78225 | Advanced |
| 6 | exp drug tolerability/ | 163354 | Advanced |
| 7 | or/3-6 | 1290145 | Advanced |
| 8 | Clinical trial/ or Randomized controlled trial/ or controlled clinical trial/ or Phase 1 clinical trial/ or Phase 2 clinical trial/ or Phase 3 clinical trial/ or Phase 4 clinical trial/ or exp Randomization/ or Single blind procedure/ or Double blind procedure/ or Crossover procedure/ or Placebo/ or Prospective Study/ or (Randomi?ed controlled trial$ or RCT or (random adj2 allocat*) or placebo$ or single blind$ or double blind$ or ((treble or triple) adj blind$)).tw. | 2954783 | Advanced |
| 9 | Clinical study/ or clinical article/ or Case-Control Studies/ or case study/ or Family study/ or Longitudinal study/ or Retrospective study/ or Cohort analysis/ or Prospective study/ or (Cohort adj (study or studies)).mp. or (Case control adj (study or studies)).tw. or (follow up adj (study or studies)).tw. or (observational adj (study or studies)).tw. or (epidemiologic$ adj (study or studies)).tw. or (cross sectional adj (study or studies)).tw. or (registry or register$ or survey).ti,ab. or (real world or RWE).ti,ab. or real-life.ti,ab. or exp seroepidemiologic studies/ or (descriptive adj3 (study or studies or design or analysis or analyses)).ti,ab,kf. or ((multidimensional or (multi adj dimensional)) adj3 (study or studies or design or analysis or analyses)).ti,ab,kf. | 8272821 | Advanced |
| 10 | (single arm or single-arm or noncomparative or non-comparative).tw. | 38304 | Advanced |
| 11 | ("case study" or "case report" or "clinical case" or "clinical report" or "individual case" or "patient case").ti,ab. | 735515 | Advanced |
| 12 | or/8-11 | 10153744 | Advanced |
| 13 | 1 and 2 and 7 and 12 | 43 | Advanced |
| **Medline** | | | |
| # | Searches | Results | Type |
| 1 | (secondary immuno?deficien* or SID or (secondary adj2 deficienc*) or secondary hypogammaglobulinemia).mp. | 6568 | Advanced |
| 2 | (subcutaneous immunoglobulin or SCIG or cuvitru or hizentra or subcuvia or vivaglobin or cutaquig or evogram or gammagard or gamunex or gamastan or gammaked or gammanorm or subgam).tw. | 734 | Advanced |
| 3 | (patient reported outcome* or PRO or self reported outcome* or quality of life or health related quality of life or qol or hrql or hrqol).ti,ab. | 684664 | Advanced |
| 4 | (treatment preference or patient preference or preference for treatment or preferred treatment).ti,ab. | 12738 | Advanced |
| 5 | (treatment satisfaction or patient satisfaction or satisfaction with treatment).ti,ab. | 53048 | Advanced |
| 6 | drug tolerability.mp. | 345 | Advanced |
| 7 | or/3-6 | 739817 | Advanced |
| 8 | Clinical trial/ or Randomized controlled trial/ or controlled clinical trial/ or Phase 1 clinical trial/ or Phase 2 clinical trial/ or Phase 3 clinical trial/ or Phase 4 clinical trial/ or exp Randomization/ or Single blind procedure/ or Double blind procedure/ or Crossover procedure/ or Placebo/ or Prospective Study/ or (Randomi?ed controlled trial$ or RCT or (random adj2 allocat*) or placebo$ or single blind$ or double blind$ or ((treble or triple) adj blind$)).tw. | 1862085 | Advanced |
| 9 | Clinical study/ or clinical article/ or Case-Control Studies/ or case study/ or Family study/ or Longitudinal study/ or Retrospective study/ or Cohort analysis/ or Prospective study/ or (Cohort adj (study or studies)).mp. or (Case control adj (study or studies)).tw. or (follow up adj (study or studies)).tw. or (observational adj (study or studies)).tw. or (epidemiologic$ adj (study or studies)).tw. or (cross sectional adj (study or studies)).tw. or (registry or register$ or survey).ti,ab. or (real world or RWE).ti,ab. or real-life.ti,ab. or exp seroepidemiologic studies/ or (descriptive adj3 (study or studies or design or analysis or analyses)).ti,ab,kf. or ((multidimensional or (multi adj dimensional)) adj3 (study or studies or design or analysis or analyses)).ti,ab,kf. | 6421864 | Advanced |
| 10 | (single arm or single-arm or noncomparative or non-comparative).tw. | 20562 | Advanced |
| 11 | ("case study" or "case report" or "clinical case" or "clinical report" or "individual case" or "patient case").ti,ab. | 556515 | Advanced |
| 12 | or/8-11 | 7562325 | Advanced |
| 13 | 1 and 2 and 7 and 12 | 12 | Advanced |

## ***Table S2. Summary of studies included in this review of SCIG in patients with SID***

| **Study** | **Patients** | **Infusion characteristics** | **Efficacy and safety outcomes** | **PROs** |
| --- | --- | --- | --- | --- |
| Data for patients with SID | | | | |
| Abadeh *et al*. 2023 (ONIT case registry) (51)  Cross-sectional study  June 2020–September 2022 | N = 140  Age, median (IQR)   - 68 (61–73) years   Sex, n (%)   - Males: 58 (41.4%)   Underlying condition, n (%)   - CLL: 52 (37.1%) - Lymphoma: 33 (23.6%) - Plasma cell dyscrasias: 26 (18.6%) - Transplantation: 12 (8.6%) - Immunosuppressive therapy for autoimmune diseases: 17 (12.1%)   IgRT, n (%)   - SCIG: 131 (93.6%; switched from IVIG: 35, 26.7%) - IgGly20: 71 (58.2%) - IgPro20: 51 (41.8%) - SCIG 16.5%: 9 (6.9%) - IVIG: 9 (6.4%) | Dose, median   - 0.44 g/kg/4 weeks   Frequency   - Weekly   Treatment duration, median   - 3.0 years | IgG levels   - Median (IQR) serum IgG level increased from 3.70 (2.35, 4.85) g/L at baseline to 8.00 (6.45, 9.60) g/L after IgRT - Results of patients receiving IVIG and SCIG were reported together; most received SCIG (131 of 140)   Infection-related outcomes   - Infection rate was reduced by 82.5% in 47.4% of patients (45/95) who initially started and remained on SCIG   Safety   - Not reported | Data reported for 55.7% (78/140) of patients at baseline and at each 6–12 monthly follow-up visit  Of the 78 patients who reported their health state after IgRT, 84.6% of patients noted improvements in health state (study-specific questionnaire)  Of the 27 patients who switched from IVIG to SCIG and responded to the questionnaire, 62.9% reported their overall health status to be better and 33.3% reported overall health status to be the same after switching |
| Cinetto *et al*. 2017 (52)^a^  Retrospective single-center study  Follow-up > 6 months | N = 124  Age, mean   - 69 years   Sex, n (%)   - NR   Underlying condition, n (%)   - NHL: 38 (30.6%) - B-cell CLL: 55 (44.4%) - MM: 19 (15.3%) - Immune-mediated disorders: 12 (9.7%)   IgRT   - SCIG 16%, 16.5%, 20%, facilitated 10% (N not reported) | Dose, mean (SD)   - 0.315 (0.0647) g/kg/month   Frequency   - Every 10–14 days   Duration, mean (SD)   - 26.7 (12.3) months | IgG levels   - Similar trough IgG levels were achieved after receiving SCIG for patients with SID and those with PIDs   Infection-related outcomes   - Reduced infection rates in both PID and SID groups   Safety   - Low number of mainly local AEs - No serious AEs reported | HRQoL (SF-36) and treatment satisfaction (TQSM) scores in SID were similar to those in PID |
| Compagno *et al*. 2014 (9)  Retrospective analysis  Follow-up NR | N = 61  Age, mean   - 67.7 years   Sex, n (%)   - 35 (57%) males   Underlying condition, n (%)   - B-cell CLL: 40 (66%) - NHL: 21 (34%) - Previously received rituximab: 42 (69%)   IgRT, n (%)   - SCIG: 61 (100%; switched from IVIG: 33, 54.1%) - SCIG 16% (Subcuvia^®b^: 35 (57.4%) - IgPro20: 26 (42.6%; switched from SCIG 16% [Vivaglobin^®b^]) | Dose   - Mean 0.075 g/kg/week   Administration   - Patients received instruction and were then able to administer SCIG independently or with help from a relative | IgG levels   - Mean (SD) serum IgG trough levels increased from baseline (3.80 [1.19] g/L) during IgRT treatment - Increase in trough IgG was significantly higher during the period of SCIG treatment than during IVIG (6.60 [1.73] g/L vs 4.74 [1.16] g/L, respectively; *p* < 0.05).   Infection-related outcomes   - Annual rate of serious bacterial infection was 0.11 per patient-year in SCIG cohort compared with 0.10 in 33 patients during IVIG treatment and 0.46 per patient-year in the 12 months prior to beginning IgRT - Reduced need for antibiotics during SCIG compared with IVIG (1.43 vs 1.82 cycles of antibiotics per patient year) and the 12 months before IgRT (2.35 cycles of antibiotics per patient year)   Safety   - The majority of AEs were mild or moderate - Short-lasting infusion-site reactions were most common, with no systemic or clinically relevant AEs - SCIG was well tolerated; 2 patients returned to IVIG - SCIG had a better safety profile than IVIG; a greater proportion of patients never complained of local AEs while receiving SCIG than when receiving IVIG (82% vs 55%) | Improvement in HRQoL (study-specific questionnaire) in most patients who switched from IVIG to SCIG (n = 28/33) |
| Grywalska and Rolinski, 2016 (53)^a^  Observation study  Follow-up 5 years | N = 20  Age, median   - 64.84 years   Sex, n (%)   - NR   Underlying condition, n (%)   - CLL: 20 (100%)   IgRT, n (%)   - SCIG: 20 (100%) | NR | IgG levels   - Median trough IgG level increased from 5.26 g/L during the last year of IVIG treatment to 6.74 g/L during SCIG (*p* < 0.001) for patients with whom SCIG dose was maintained/reduced compared with IVIG.   Infection-related outcomes   - Annual rate of infection was lower during SCIG than IVIG (32 vs 69, respectively, *p* = 0.004).   Safety   - 8 treatment-related local AEs were reported during study period | Significant improvements in QoL (PRO measure(s) not reported) on switching to SCIG |
| Innocenti *et al*. 2022 (54)  Observational study  October 2019–December 2020 | N = 10  Age, mean (range)   - 66 (56–88) years  Sex, n (%)  - NR   Underlying condition, n (%)   - CLL: 10 (100%)  Comorbidities (n)Thyroiditis (1), hypertension (4), diabetes mellitus (4), lung diseases (5) IgRT, n (%)   - SCIG: 10 (100%) | Dose   - Total dose of 10 g via infusion pump every 15 days  Administration  - First dose administered in hospital, subsequent doses administered at home | IgG levels   - Median IgG levels increased from 4.85 g/L before receiving SCIG to > 6.00 g/L from 6 months onward  Infection-related outcomes  - No patient experienced an infectious event during the study.   Safety   - All patients tolerated the therapy well - No patients required treatment to be interrupted - No changes in dose or administration schedule were required - One patient presented with CTCAE 4.0 grade 2 atopic dermatitis | Improvement in QoL in all patients (PRO measure(s) and periods of assessment not reported) |
| Keith *et al*. 2022 (CANCUN) (55)  Phase 4, non-interventional, prospective, single-arm study  NCT03716700  Follow-up 12 months | N = 64  Age, median (range)   - 69 (48–83) years   Sex, n (%)   - Males: 28 (43.8%)   Underlying condition, n (%)   - SID: 65 (52.0%)^c^ - CLL: 36 (55.4%) - MM: 4 (6.2%) - Post-allogenic HSCT: 1 (1.5%) - Other: 24 (36.9%)   IgRT, n (%)   - SCIG IgGly20: 64 (100%) | Dose   - Median (IQR) weekly dose 8.0 (6.0–10.0) g   Frequency   - Most patients with SID (n = 47, 87%) infused once weekly   Volume   - At 12 months, the median (IQR) infusion volume was 40 (35–50) mL   Treatment duration, median (IQR)   - 60 (40–65) minutes   Administration   - 38 patients (59.4%) used an infusion pump rather than manual administration | IgG levels   - Median (IQR) IgG trough levels for patients with SID after 12 months of SCIG treatment were 8.3 (7.9, 9.0) g/L (n = 55), compared with 8.2 (7.4, 10.7) g/L at initiation (n =13)   Infection-related outcomes (combined with patients with PID, total n = 125)   - Bacterial infections were reported by 19.8% of all patients at 12 months post-initiation   - Acute serious bacterial infections occurred in 4 patients in the SID group, and 2 in the PID group   Safety   - Most AEs were mild or moderate in severity   - 3 severe AEs occurred in patients with SID - Serious AEs were reported in 4 patients (6.3%), none were considered to be related to SCIG | Generally high treatment satisfaction (TSQM-9 and LQI) and QoL (TPQ) at 12-months after SCIG initiation  Attributes of SCIG that were liked included frequency and ease of administration, and the option of self-administration. 9.4% of participants disliked the number of punctures per month. |
| Mustafa *et al*. 2020 (56)  Proof of concept prospective case series  Follow-up 52 weeks | N = 15  Age, median (IQR)   - 71 (64.5–74.5) years   Sex, n (%)   - Males: 8 (53.3%)   Underlying condition, n (%)   - Follicular lymphoma: 6 (40.0%) - Diffuse large B-cell lymphoma: 5 (33.3%) - Lymphoplasmacytic lymphoma: 2 (13.3%) - Mantle cell lymphoma: 1 (6.7%) - Marginal zone lymphoma: 1 (6.7%)   IgRT, n (%)   - SCIG IgGly20: 10 (66%) - No IgRT: 5 (33%) | Dose   - Starting dose of 0.1 g/kg/week for a 52-week period   Duration, range   - 45–90 minutes   Administration   - Patients received instruction and were then able to administer SCIG independently | In the 9 patients who completed 52 weeks of treatment:  IgG levels   - Median (IQR) IgG increased from 5.77 (4.78, 6.25) g/L at baseline to 10.22 (9.98, 11.15) g/L after 52 weeks (*p* = 0.008)   Infection-related outcomes   - Annualized non-neutropenic infection rate reduced to 6 during SCIG treatment, compared with 17 in the 12 months before   Safety and tolerability   - SCIG was well tolerated - No patients experienced any systemic AEs during the study period | HRQoL (SF-36) scores recorded every 28 ± 7 days did not change significantly during the study period; many patients had high scores at enrollment |
| Mustafa *et al*. 2021 (57)^d^  Exploratory proof of concept prospective case series  NCT03730129  Follow-up 9 months | N = 15  Age, median (IQR)   - 69 (67–75) years   Sex, n (%)   - 13 (86.7%) males   Underlying condition, n (%)   - CLL: 15 (100%)   IgRT   - SCIG IgPro20: 10 (100% of patients who received treatment) | Dose   - Fixed dose of 0.13 g/kg/week - Median (IQR) weekly dose was 11.5 (11–12) g   Duration   - Median (IQR) 61 (56–64) minutes   Administration   - Patients received instruction and were then able to administer SCIG independently at home. - Median number of infusion sites was 2.5 | In the 9 patients who completed 24 weeks of treatment:  IgG levels   - Median (IQR) IgG increased from 6.7 (5.7–8.2) g/L to 10.5 (10.4–11.7) g/L after 24 weeks of SCIG treatment   Infection-related outcomes   - The number of non-neutropenic infections decreased from 14 in the 6 months prior to IgRT to 5 during 6 months of IgRT   Safety   - No localized or systemic reactions - One patient discontinued treatment owing to fatigue symptoms | HRQoL (SF-36) scores recorded every 28 ± 7 days did not change significantly during the study period; many patients had high scores at enrollment |
| Vacca *et al*. 2018 (58)  Prospective, controlled, randomized study | N = 46  Age, mean (range)   - 71 (56–85) years   Sex, n (%)   - Males: 25 (54.3%)   Underlying condition, n (%)   - MM: 46 (100%)   IgRT, n (%)   - SCIG IgPro20: 24 (100% of treatment arm) - No SCIG: 22 (100% of control arm) | Dose   - Monthly total dose 0.4-0.8 g/kg divided into weekly doses   Administration   - Infusion pump; abdomen or anterior thigh | IgG levels   - Median trough IgG levels 8.3–9.5 g/L in SCIG cohort compared with 2.4–5.2 g/L in no-SCIG cohort   Infection-related outcomes   - Severe infections lower in the treatment arm vs the control arm (*p*< 0.01) - 8 vs 121 mean days/year hospitalization due to severe infection in SCIG and control cohorts (p < 0.001) - 28 vs 217 mean days with antibiotic treatment for SCIG cohort vs control cohort (p < 0.001)   Safety   - AEs were limited to the infusion site and the majority were mild - 3 patients discontinued treatment owing to AEs; 2 patients experienced pain and inflammation at the infusion site, one patient experienced an allergic skin reaction that resolved with steroid and anti-histamine treatment | Higher proportion of patients with positive HRQoL (SF-36) scores in the SCIG cohort vs the control cohort. |
| Windegger *et al*. 2019 (59)^a^  Pilot study  12 months of IVIG followed by 12 months of SCIG | N = 13  Age, mean   - 63 years   Sex, n (%)   - NR   Underlying condition   - NR   IgRT, n (%)   - SCIG: 13 (100%) - IVIG: 13 (100%) | Dose   - Fixed dose of 0.1 g/kg/week   Administration   - Mean of 3 training sessions required to reach competency for SCIG administration | IgG levels   - Mean serum trough levels 8.4 g/L during SCIG vs 7.1 g/L during IVIG (*p* = 0.009)   Infection-related outcomes   - Mean annual infection rate 2.15 for SCIG vs 1.62 for IVIG - Hospitalizations due to infection decreased on SCIG vs IVIG   Safety   - The majority of patients experienced infusion-site reactions, which were predominantly considered as manageable - AEs tended to be lower with SCIG compared with IVIG | No improvements in health and QoL (study-specific questionnaire) after switching from IVIG to SCIG |
| Windegger *et al*. 2021 (60)  Longitudinal observational study  3 years follow-up on SCIG | N = 17  Age, median (range)   - 63 (53–76) years   Sex, n (%)   - Males: 7 (41.2%)   Underlying condition, n (%)   - NHL: 9 (52.9%) - CLL: 5 (29.4%) - MM: 2 (11.8%) - HL: 1 (5.9%)   IgRT, n (%)   - SCIG: 17 (100%; switched from IVIG: 13, 76.5%) | Dose   - Initiated at 0.4/g/kg/month split into weekly doses - Mean (SD) grams per patients (32.5 [9.07] g/patient during the first year of SCIG treatment, 32.09 [8.82] during the third year)   Administration   - Patients received instruction and were then able to administer SCIG independently | IgG levels   - Mean (SD) IgG level on SCIG (n = 17) were 8.0 (1.75) g/L at 1 year, 8.7 (2.75) g/L at 2 years, 7.6 (2.89) g/L at 3 years, compared with 7.0 (2.77) g/L in 13 patients on IVIG, prior to switching to SCIG   Infection-related outcomes   - Mean (SD) infection rate during SCIG ranged from 1.58 (1.54)–2.06 (1.52), respectively, compared with 2.08 (2.14) in 13 patients on IVIG, prior to switching to SCIG   Safety   - All patients reported SCIG AEs as “always manageable” - Most common AEs were swelling, redness, and pain at the infusion site - There were no systemic AEs | Improved QoL (study-specific questionnaire) reported by 75% of patients at 12 months after switching from IVIG. |

^a^Congress abstract.

^b^Discontinued: Subcuvia, Baxter; Vivaglobin, CSL Behring.

^c^One patient had both PID and SID

^d^Article identified from hand searches.

AE, adverse event; CLL, chronic lymphocytic leukemia; CTCAE, Common Terminology Criteria for Adverse Events; HL, Hodgkin’s lymphoma; HRQoL, health-related quality of life; HSCT, HSCT, hematopoietic stem cell transplantation; IgG, immunoglobulin G; IgRT, immunoglobulin replacement therapy; IQR, interquartile range; IVIG, intravenous immunoglobulin; LQI, Life Quality Index; MM, multiple myeloma; NHL, non-Hodgkin’s lymphoma; NR, not reported; PID, primary immunodeficiency disease; PRO, patient-reported outcome; QoL, quality of life; SCIG, subcutaneous immunoglobulin; SD, standard deviation; SEM, standard error of the mean; SF-36, 36-item Short Form Health Survey; SID, secondary immunodeficiency; TPQ, treatment preference questionnaire; TSQM-9, Treatment Satisfaction Questionnaire for Medication-9.

## ***Table S3. Summary of IgRT dose and IgG level in studies which compare IVIG and SCIG.***

| **Study** | **During IVIG treatment** | **During SCIG treatment** |
| --- | --- | --- |
| Data for patients with SID | | |
| Compagno *et al*. 2014 (9)  Retrospective analysis  Follow-up NR | N = 33  Dose   - Mean 0.300 g/kg/month   IgG level   - Mean (SD) trough 4.74 [1.16] g/L | N = 61  Dose   - Mean 0.075 g/kg/week   IgG level   - Mean (SD) trough 6.60 [1.73] g/L |
| Grywalska and Rolinski, 2016 (53)^a^  Observation study  Follow-up 5 years | N = NR  Dose   - NR^b^   IgG level   - Mean trough 5.26 g/L | N = 20  Dose   - NR^b^   IgG level   - Mean trough 6.74 g/L |
| Windegger *et al*. 2019 (59)^a^  Pilot study  12 months of IVIG followed by 12 months of SCIG | N = 13  Dose   - 0.4 g/kg/month   IgG level   - Mean trough 7.1 g/L | N = 13  Dose   - Fixed dose of 0.1 g/kg/week   IgG level   - Mean trough 8.4 g/L |
| Windegger *et al*. 2021 (60)  Longitudinal observational study  3 years follow-up on SCIG | N = 13  Dose   - Mean (SD) 30.2 (6.83) g/patient   IgG level   - Mean (SD) 7.0 (2.77) g/L | N = 17  Dose   - Initiated at 0.4/g/kg/month split into weekly doses - Mean (SD) 32.5 (9.07) g/patient during the first year of SCIG treatment, 32.09 (8.82) g/patient during the third year   IgG level   - Mean (SD) was 8.0 (1.75) g/L at 1 year, 8.7 (2.75) g/L at 2 years, 7.6 (2.89) g/L at 3 years |

^a^Congress abstract.

^b^SCIG dose was maintained/reduced compared with IVIG.

IgG, immunoglobulin G; IgRT, immunoglobulin replacement therapy; IVIG, intravenous immunoglobulin; NR, not reported; SCIG, subcutaneous immunoglobulin; SD, standard deviation; SID, secondary immunodeficiency.

## ***Figure S1. Study selection.***


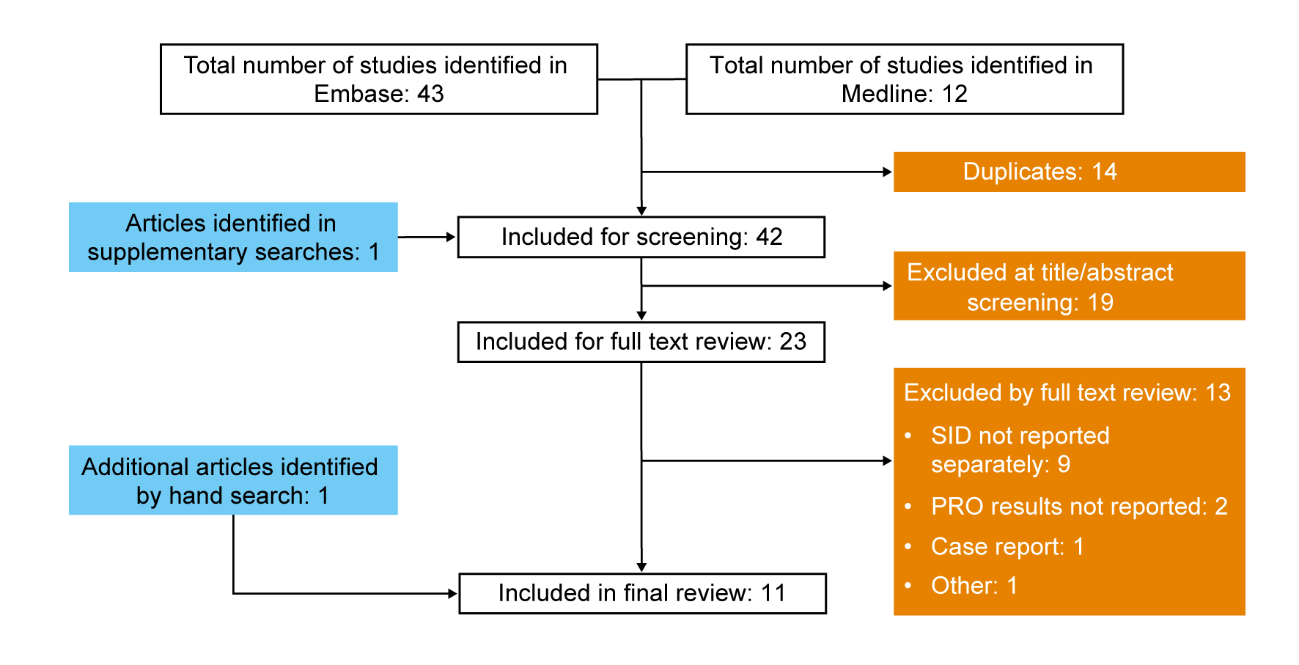


Reasons for exclusion at screening were: duplicate publication, hyaluronidase-facilitated SCIG and any indication other than SID.

PRO, patient-report outcomes; SCIG, subcutaneous immunoglobulin; SID, secondary immunodeficiency.
